# Preclinical Therapeutic Potential of a Nitrosylating Agent in the Treatment of Ovarian Cancer

**DOI:** 10.1371/journal.pone.0097897

**Published:** 2014-06-02

**Authors:** Shailendra Giri, Ramandeep Rattan, Mandar Deshpande, Jacie L. Maguire, Zachary Johnson, Rondell P. Graham, Viji Shridhar

**Affiliations:** 1 Department of Experimental Pathology, Mayo Clinic College of Medicine, Mayo Clinic, Rochester, Minnesota, United States of America; 2 Department of Anatomic/Clinical Pathology, Mayo Clinic College of Medicine, Mayo Clinic, Rochester, Minnesota, United States of America; 3 Women's Health Services, Henry Ford Health System, Detroit, Michigan, United States of America; 4 Department of Neurology, Henry Ford Health system, Detroit, Michigan, United States of America; National Health Research Institutes, Taiwan

## Abstract

This study examines the role of s-nitrosylation in the growth of ovarian cancer using cell culture based and *in vivo* approaches. Using the nitrosylating agent, S-nitrosoglutathione (GSNO), a physiological nitric oxide molecule, we show that GSNO treatment inhibited proliferation of chemoresponsive and chemoresistant ovarian cancer cell lines (A2780, C200, SKVO3, ID8, OVCAR3, OVCAR4, OVCAR5, OVCAR7, OVCAR8, OVCAR10, PE01 and PE04) in a dose dependent manner. GSNO treatment abrogated growth factor (HB-EGF) induced signal transduction including phosphorylation of Akt, p42/44 and STAT3, which are known to play critical roles in ovarian cancer growth and progression. To examine the therapeutic potential of GSNO *in vivo*, nude mice bearing intra-peritoneal xenografts of human A2780 ovarian carcinoma cell line (2×10^6^) were orally administered GSNO at the dose of 1 mg/kg body weight. Daily oral administration of GSNO significantly attenuated tumor mass (p<0.001) in the peritoneal cavity compared to vehicle (phosphate buffered saline) treated group at 4 weeks. GSNO also potentiated cisplatin mediated tumor toxicity in an A2780 ovarian carcinoma nude mouse model. GSNO’s nitrosylating ability was reflected in the induced nitrosylation of various known proteins including NFκB p65, Akt and EGFR. As a novel finding, we observed that GSNO also induced nitrosylation with inverse relationship at tyrosine 705 phosphorylation of STAT3, an established player in chemoresistance and cell proliferation in ovarian cancer and in cancer in general. Overall, our study underlines the significance of S-nitrosylation of key cancer promoting proteins in modulating ovarian cancer and proposes the therapeutic potential of nitrosylating agents (like GSNO) for the treatment of ovarian cancer alone or in combination with chemotherapeutic drugs.

## Introduction

Ovarian cancer (OvCa) is the fifth most common cause of death from all cancers among women in the United States and the leading cause of death from gynecological malignancies [1]. The high mortality rates associated with OvCa is due to the majority of patients (75%) presenting with widespread (stage III or greater) disease at the time of diagnosis [2]. The poor 5-year survival (30%) is due to the fact that most OvCa’s are inoperable at the time of diagnosis. If detected early, more than 90% of the patients have a better prognosis and respond to therapy compared to patients with an advanced stage of the disease. Consequently, a better understanding of key molecular events playing important roles in OvCa may lead to better diagnosis and treatment.

S-nitrosoglutathione (GSNO) is a nitrosylating agent which mediates the posttranslational process of S-nitrosylation on proteins resulting in modulation of their activity. S-nitrosylation is an important biological reaction of nitric oxide (NO) and refers to the conversion of thiol groups, including cysteine residues in proteins, to form S-nitrosothiols. It is a mechanism for dynamic posttranslational regulation of most or all major classes of protein and is independent of enzyme catalysis, labile modification, on/off switch-like photophosphorylation [3]; however, denitrosylation can be enzymatic or non-enzymatic. An increasing number of proteins have been found to undergo S-nitrosylation *in vivo,* called S-nitrosothiols, and they play an important role in various processes ranging from signal transduction, DNA repair, host defense, and blood pressure control to ion channel regulation and neurotransmission [3]. However, the role of S-nitrosylation in OvCa growth and progression has not been studied.

This study was planned to examine the therapeutic effect of a nitrosylating agent (GSNO) in OvCa using *in vitro* and *in vivo* models and to examine the role of nitrosylation in OvCa.

## Methods

### Reagents and antibodies

GSNO was purchased from World Precision Instruments (Sarasota, FL) and its purity is <98%. The following antibodies phospho-STAT3 (Y705) (Cat # 9145, used at 1∶1000), pAkt (Ser473) (Cat # 4060, used at 1∶1000), p-p42/44 (Thr202/Tyr204) (Cat # 4370, used at 1∶1000), STAT3 (Cat # 9139, used at 1∶1000), Akt (Cat # 4685, used at 1∶1000), p42/44 (Cat # 9107, used at 1∶1000) were from Cell Signaling (Danvers, MA). Beta-actin (b-actin) was purchased from Sigma (St. Louis, MO). Fetal bovine serum was purchased from BioAbChem (Ladson, SC). Recombinant STAT3 was purchased from SignalChem (Richmond, Canada).

### Cell Culture

Human OvCa cell line SKOV3 and OVCARs were from American Type Culture Collection (Manassas, VA). A2780, C200 and OVCAR4 cell lines were a kind gift from Dr. Tom Hamilton (Fox Chase Cancer Center) [4]. PE01 and PE04 were a kind gift from Dr. Taniguchi (University of Washington, Seattle) [4]. All OvCa cell lines were maintained and cultured in complete Roswell Park Memorial Institute (RPMI) media containing 10% fetal bovine serum and antibiotics.

### Animals

#### Ethics Statement

Six- to eight-week old female nude mice were purchased from the National Cancer Institute-Frederick Cancer Research and Development Center (Frederick, MD). All mice were housed and maintained under specific conditions in facilities at the Mayo Clinic in Rochester, MN. The facilities are approved and inspected by the American Association for Accreditation of Laboratory Animal Care (AAALAC Accreditation # 000717) and in accordance with current regulations and standards of the U.S. Department of Agriculture, U.S. Department of Health and Human Services, and the National Institutes of Health. All studies were approved and supervised by the Mayo Clinic Institutional Animal Care and Use Committee (IACUC) under the protocol number A13909.

Mice were maintained according to Institutional IACUC approved protocol. A2780 cells were washed twice and resuspended in phosphate buffered saline (PBS) at 2×10^6^/100 µl and injected into the intraperitoneal cavity of the nude mice (day 0). Treatment with GSNO (1 mg/kg of body weight) was started 3 days after the inoculation of the cells. GSNO was administered by oral gavage in 0.1 mL volume using a 20 gauge feeding needle with ball diameter of 2.25 mm (Braintree Scientific, MA). The control group received PBS as a vehicle. The mice were monitored daily for any discomfort and weighed every third day to check for tumor growth. For combination studies, cisplatin treatment (4 mg/kg of body weight) by intraperitoneal injections was given on days 7, 14 and 21 along with GSNO treatment as described above (day 0 was taken as the day of inoculation of cells). After tumor induction, mice were monitored daily for signs of any distress. Mice were humanly killed with overdose of CO2 at 4 weeks when the tumor burden reached the mandate weight in untreated mice [4], [5] and tumors were excised and fixed in formalin for sectioning.

### Immunoblot analysis

After the stipulated time of incubation in the presence or absence of indicated amounts of GSNO, immunoblot analysis with specific antibodies was performed as previously described [6]–[9]. In brief, treated and untreated A2780 or SKOV3 cells were treated with HB-EGF (50 ng/ml) and/or GSNO (2 h pretreatment prior to the addition of HB-EGF on cells) at various time periods (5–20 min) were lysed in lysis buffer [50 mM Tris-HCl (pH 7.5), 250 mM NaCl, 5 mM EDTA, 50 mM NaF, 1 mM DTT, 50 mM Na3VO4 and 0.5% Nonidet P-40] containing a protease inhibitor cocktail (Sigma, St. Louis, MO). Forty μg of proteins were resolved by SDS-PAGE and transferred onto nitrocellulose membrane. The membrane was then blocked for 1 hour in 5% nonfat dry milk TTBS (20 mM Tris, 500 mM NaCl, and 0.1% Tween 20, pH 7.5) and incubated overnight in primary antisera against phosphor-STAT3 (Y705), STAT3, p-p42/44 (Thr202/Y204), p42/44, pAkt (Ser473), Akt or β-actin containing 5% nonfat dry milk or 5% BSA in case of phospho-antibodies. After incubation with HRP-conjugated secondary Ab, blots were developed with an ECL detection system (GE Healthcare, Piscataway, NJ).

### Proliferation assays

Cells (2.5–5.0×10^4^) were plated in 24-well plate in triplicates and treated with indicated concentrations of GSNO for 48 hours. Inactive oxidized GSNO was used as a control. Inactive oxidized GSNO was prepared by exposing GSNO solution (0.2 mM in DMSO) to light for 7 days. Oxidized GSNO is colorless and does not produce NO when added to cell media alone or with cells unlike unexposed GSNO (**Figure S1A & C**). MTT assay was performed as previously described [8] to ascertain the number of live cells.

### Colony Formation Assay

Cells (2×10^3^) were plated in triplicates in 6-well plate and after 24 hour, cells were treated with indicated concentrations of GSNO once. The cells were allowed to form colonies for up to 2 weeks and complete media was replaced every fourth day. Colonies were stained with MTT and counted as previously described [8].

### Dose-effect analyses

The concentration for 50% inhibition (IC 50) was determined on the basis of dose-response curves from MTT assay and was calculated using CalcuSyn software (Biosoft, Cambridge, UK).

### Migration and Invasion Assay

SKOV3 cells were grown in serum-free media overnight. Scratch migration and invasion assays were measured as described before [5]. For wound closure assay, SKOV3 cells were grown to confluence in 24-well plates and kept in low serum conditions (0.2%) overnight. A scratch was created in the middle of the well with a sterile 200 µl tip, and media replaced with the respective treatments. Micrographs were collected at 0 and 24 hour. The pixel distance between the gaps was measured at a pre-determined point at each time point. The distance covered was subtracted from the start time point to estimate the distance covered by the migrating cells [10]. For invasion assay, SKOV3 cells in suspensions (500 µl, 2.5×10^4^) were seeded on the top of Matrigel-coated transwell plates (8- μM pore diameter; BD Biosciences). The lower chambers contained serum-free media containing various growth factor including HB-EGF and SDF1 at the concentration of 25 ng/ml. Cells invading the lower surface of the filter coated with a thin layer of Matrigel were stained with 0.5% crystal violet (60% PBS, 40% EtOH) and counted with an inverted microscope. The results from at least two independent experiments in triplicates are presented.

### Immunohistochemistry (IHC)

The tumors excised from mice were fixed in 10% paraformaldehyde for 48 hours and paraffin embedded. Four-μm-thick consecutive sections were cut and processed for immunohistochemistry for CD31 (Cat # sc31045, used at 1∶100) and Ki-67 (Cat # M7240, used at 1∶100). Solutions obtained from Dako Cytomation (Glostrup, Denmark) were used for performing immunostaining. In brief, tissue sections were deparaffinized, unmasked, blocked with avidin-biotin and incubated with primary antibody overnight. Next day the reaction was detected by using chromogen according to the manufacturer’s instructions (Dako, Glostrup, Denmark). The positive cells stained brown. The slides were examined under light microscope and representative pictograms were taken from a minimum of 5 or 6 different slides of each group. For CD31 staining, FITC-labeled secondary antibody was used and visualized using fluorescent microscope in 6 sections per group.

### Mitotic count and Live Tumor Measurements

The mitotic count was recorded on hematoxylin and eosin stained sections using the Olympus BX-41 light microscope at high power field (HPF; ×400). Cells undergoing mitosis were counted in the tumors, in the most active area (“hot spots”) in a minimum of 5 consecutive HPFs. The average number of cells undergoing mitosis per HPF was enumerated. Maximum diameter of viable tumor was calculated by summing the largest unidimensional diameter of each fragment of tumor using the Olympus BX-41 microscope and a micrometer. Similarly, necrotic areas were measured and the composite live tumor size was calculated from each slide as published before [4], [5].

### Statistical Analysis

Data are expressed as the means + SD and were statistically analyzed using Student’s t-test (Prism). P<0.05 was considered to be statistically significant.

## Results

### GSNO inhibits proliferation of OvCa cell lines

**Figure 1:**
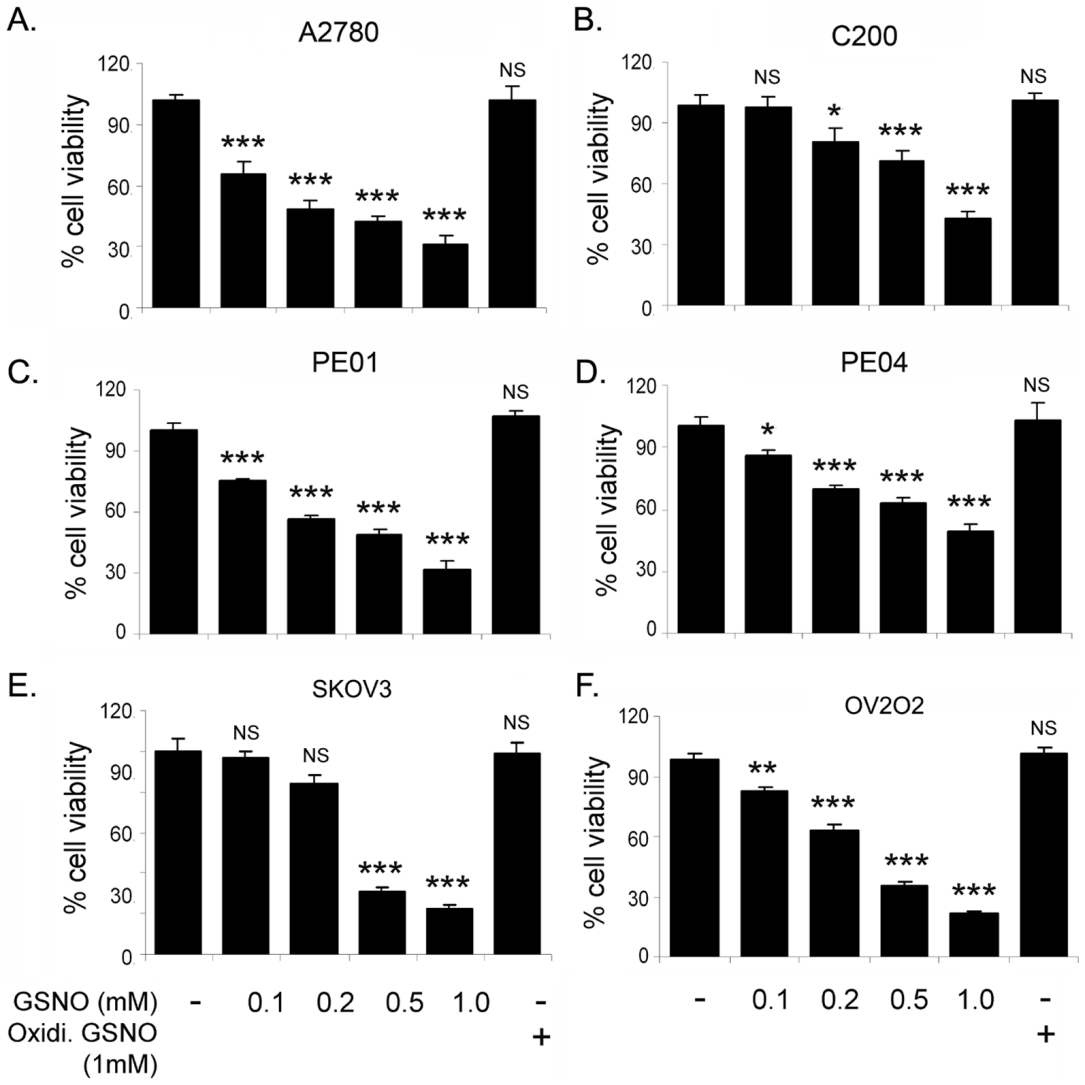
GSNO attenuates cell proliferation in ovarian cancer cell lines. Percentage viability of A2780, C200, PE01, PE04, SKOV3 and OV202 treated with indicated doses of S-nitrosoglutathione (GSNO; 0.1–1 mM) was determined by MTT assay. Inactive GSNO (oxidized, last bar) was used as a control. The data represents 3 individual experiments done in triplicate. ***p<0.001; **p<0.01; *p<0.05 and NS; not significant compared to untreated cells using Student’s t-test (Prism).

To assess the effect of GSNO treatment on OvCa growth, various OvCa cell lines (A2780, C200, PE01, PE04, OV202 and SKOV3) were treated with various concentrations of GSNO (0.1–1 mM). Proliferation was determined by MTT assay after 24 hours. Treatment with GSNO inhibited the proliferation of these OvCa cell lines significantly in a dose-dependent manner (**Fig. 1**) including cisplatin resistant C200 (**Fig. 1B**), taxol resistant PE04 (**Fig. 1D**) and aggressive SKOV3 cell lines (**Fig. 1E**). Addition of GSNO in media released NO in dose dependent manner; however, oxidized GSNO did not produce NO (**Fig. S1B–C)**. We also measured IC50 of GSNO on cell proliferation of various OVCA cell lines using CalcuSyn program. IC50 for GSNO were 0.162, 0.385, 0.337, 0.427, 0.196 and 0.235 mmol/L in A2780, C200, PE01, PE04, OV2O2 and SKOV3, respectively **(Table S1)**. GSNO treatment also attenuated cell proliferation of other OvCa cell lines including OVCAR-3, -4, 5, -7, -8 and -10 in a dose dependent manner (**Fig. S2**). The IC50 for GSNO in these cell lines were found differentially and listed in table S1. The inactive oxidized form of GSNO had no effect on the proliferation of OvCa cell lines.

**Figure 2:**
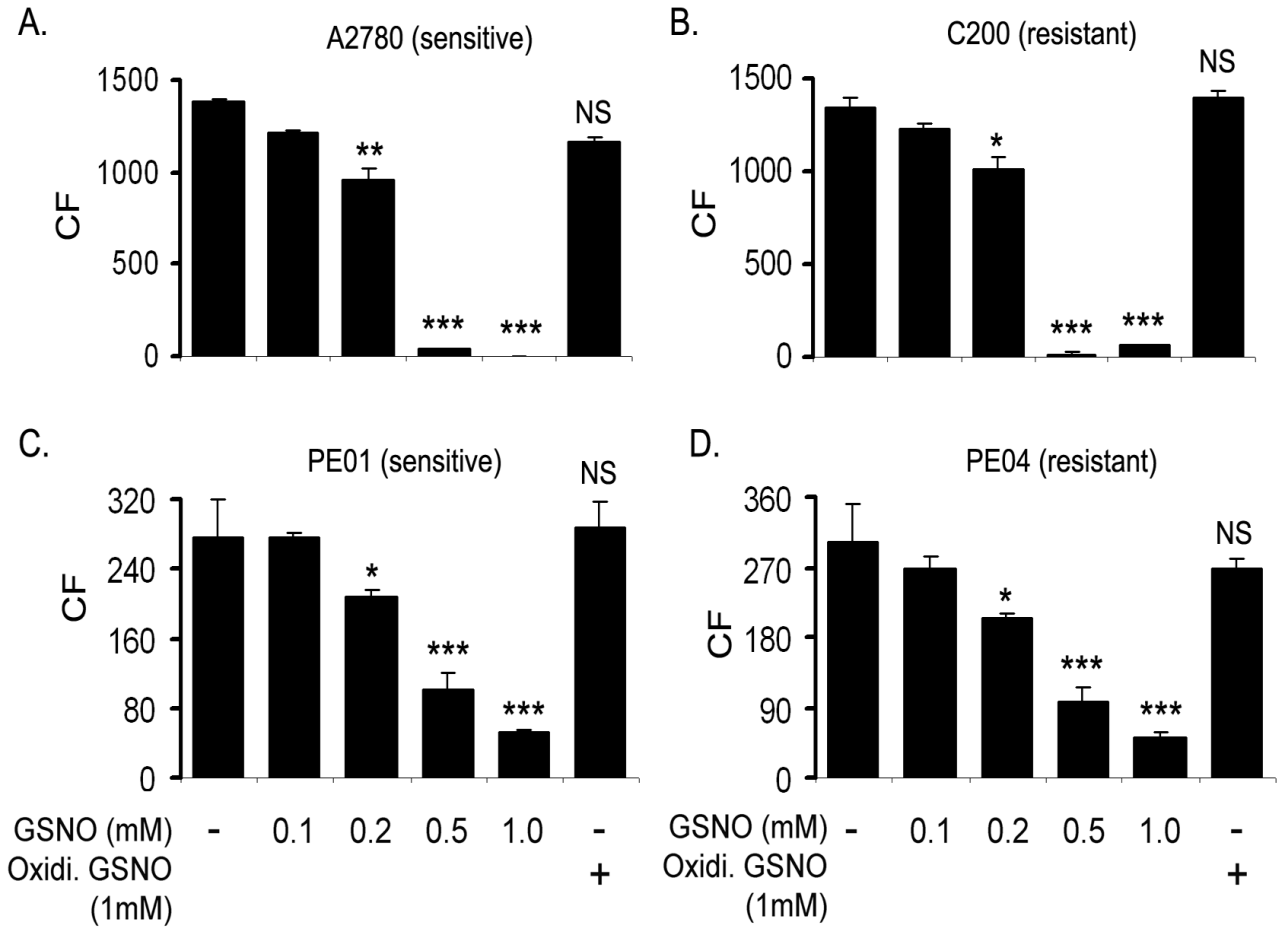
GSNO inhibits colony formation in chemosensitive and chemoresistant ovarian cancer cell line. Cells (2×10^3^)/well (A2780, C200, PEO1 and PEO4) were plated in 6-well plates and treated with indicated concentrations of S-nitrosoglutathione (GSNO) once. Oxidized GSNO was used as a negative control (last bar). After 2 weeks, colonies were stained with MTT and counted. Results are shown as mean ± SD of triplicates. *p<0.05, **p<0.01, ***p<0.001 and NS; not significant compared to untreated cells using Student’s t-test (Prism).

To determine if this inhibition was reflected in clonogenic survival, the colony forming ability of A2780, C200, PE01 and PE04 cells were performed. As shown in **Figure 2**, a single treatment of GSNO significantly attenuated clonogenic survival of these OvCa cell lines in a dose dependent manner compared to untreated cells (**Fig. 2**). IC50 for GSNO was found to be 0.122, 0.161, 0.356 and 0.352 mmol/L in in A2780, C200, PE01 and PE04, respectively **(Table S2)**. Inactive oxidized form of GSNO had no effect on the colony formation of OvCa cell lines. These results suggest that GSNO treatment can result in a sustained inhibition of proliferation of various OvCa cell lines, including chemoresistant cell lines (C200 and PE04) (**Fig. 2B, C & F**).

### GSNO attenuates growth factors induced cell migration and invasion *in vitro*

**Figure 3:**
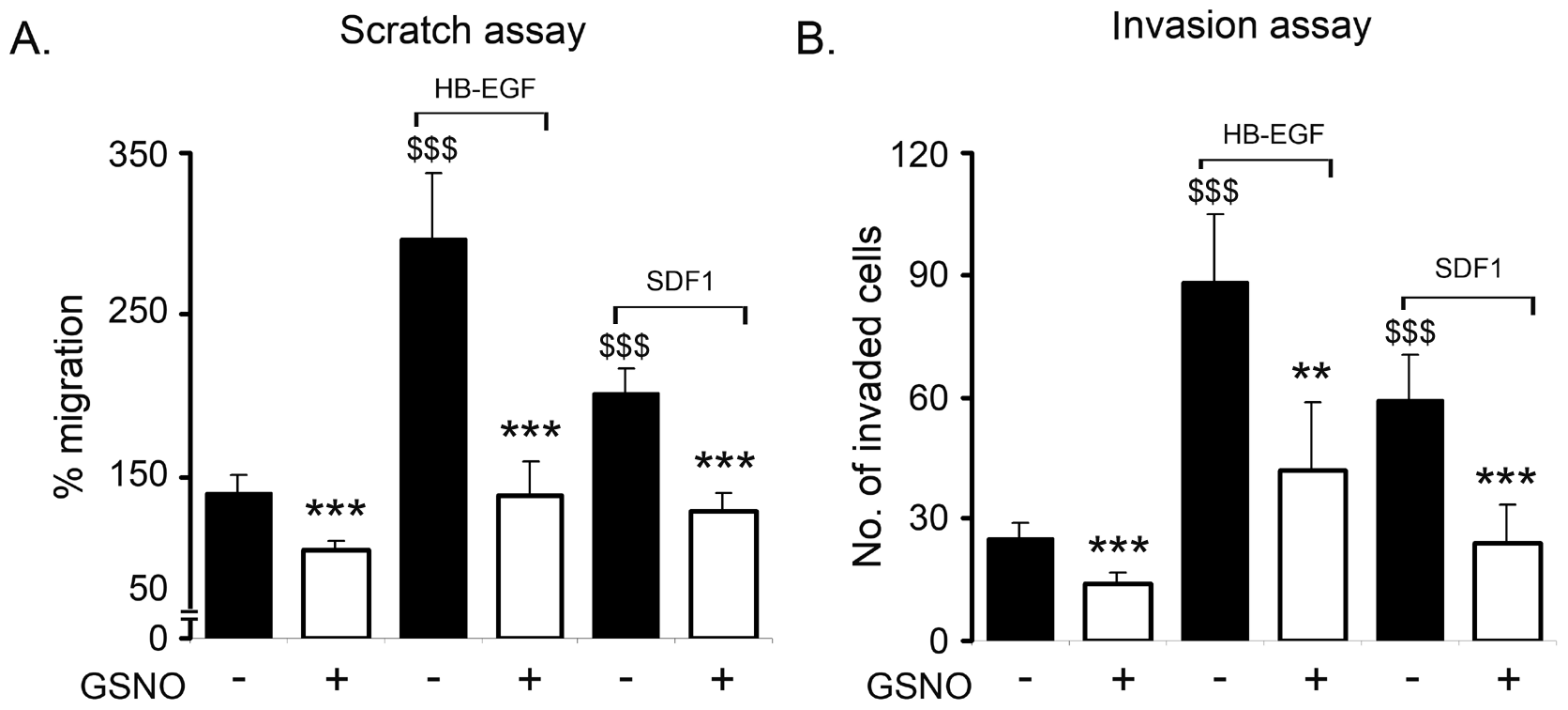
GSNO treatment attenuated cell migration and invasion in ovarian cancer cell line. **A**. Cells grown with HB-EGF and SDF1 in absence or presence of S-nitrosoglutathione (GSNO) and cell migration were measured by wound closure assay as described in the material and methods. Results are shown as mean ± SD of n = 7. **B.** To examine the effect of GSNO on invasion, 1×10^5^ SKOV3 cells were seeded into the upper wells with 0.2 mM GSNO in the upper chamber of transwell in 500 µl culture medium. Various growth factors (HB-EGF and SDF1) (25 ng/ml) was added to the underlying media. Twenty-four hours later, invasion was determined following manufacturer’s instructions. Results are shown as mean ± SD of triplicates. $$$ p<0.001 growth factor treated compared to control. *p <0.05; ***p<0.001 GSNO treated compared to growth factor using Student’s t-test (Prism).

We next examined the effect of GSNO on growth factor mediated cell migration and invasion. SKOV3 cells grown overnight in low serum (0.2%) containing medium were scratched using a sterile 200 µl pipette tip, once they had reached 90% confluency. Various growth factors including HB-EGF and SDF1 (50 ng and 25 ng/ml, respectively) were added individually to the medium in the presence or absence of GSNO (200 µM). Twenty-four hours later, the rate of wound closure was calculated. As shown in **Figure 3A**, GSNO inhibited all HB-EGF and SDF1 mediated cell migration in SKOV3 cell line. Similar results were obtained for A2780 and C200 cell lines (data not shown). Next we assessed the effect of GSNO in modulating growth factor mediated invasion of SKOV3 cells using Boyden chamber migration assay (BD Bioscience, San Jose, CA) as previously described [11]. **Figure 3B** clearly demonstrates that GSNO treatment significantly inhibited growth factor induced invasion of SKOV3 cells compared to untreated cells. These results indicate that GSNO has the ability to inhibit the migration and invasion of OvCa cells.

### GSNO abrogates growth factor induced signaling in OvCa cells

**Figure 4:**
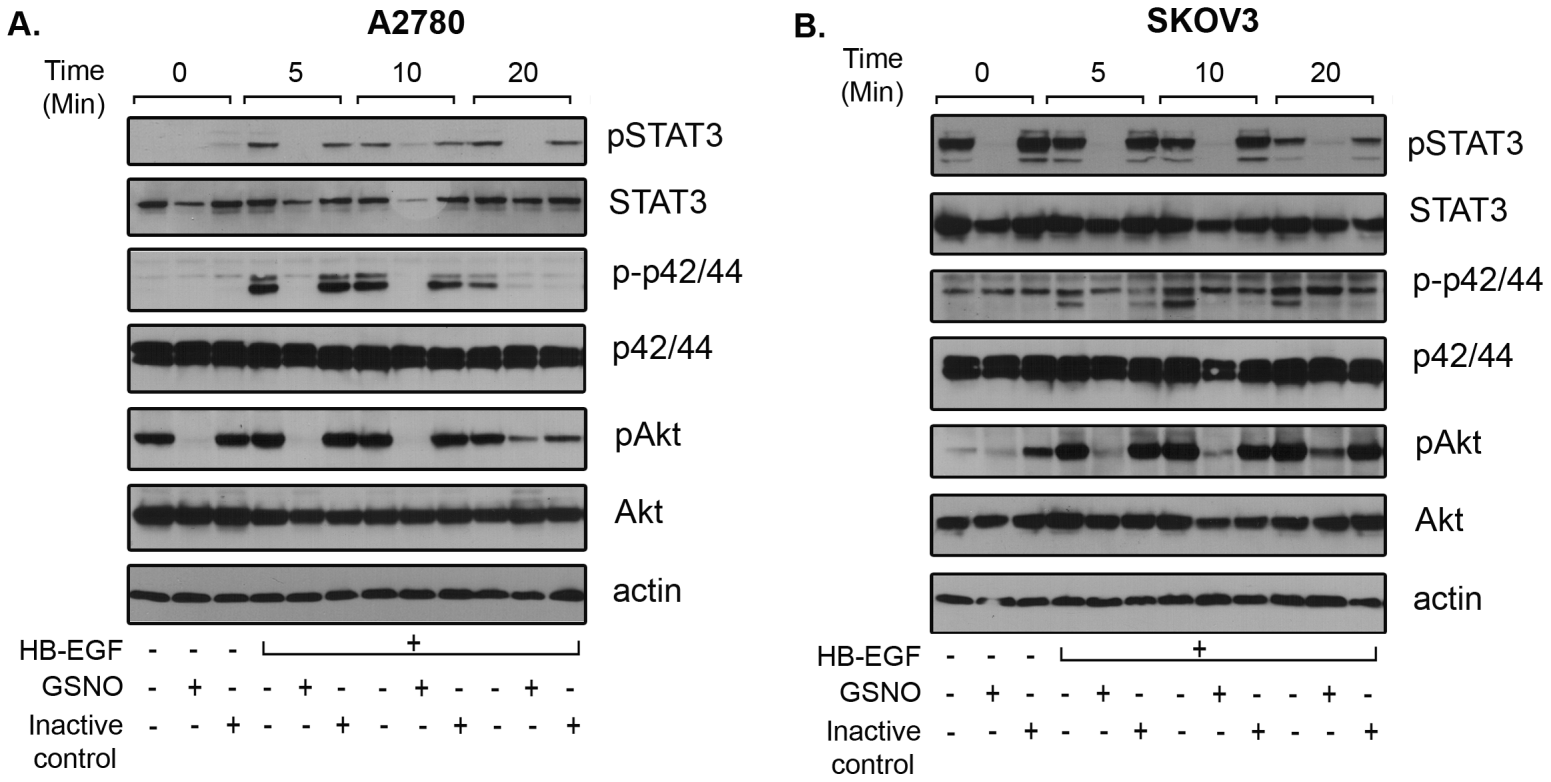
GSNO treatment attenuates STAT3 activation and proliferative signaling in ovarian cancer cell lines. A2780 (**A**) and SKOV3 (**B**) cells were plated, serum starved overnight and treated with S-nitrosoglutathione (GSNO; 0.5 mM) or inactive control (oxidized GSNO; 0.5 mM) in the presence or absence of HB-EGF (50 ng/ml) for various time periods (5–20 min). Cells were harvested at indicated time points and processed for the detection of various signaling molecules including pSTAT3 (Tyr705), pAkt (Ser473) and p-p42/44 (Thr202/Tyr204) using their specific antibodies from Cell Signaling (Danvers, MA). Total STAT3, Akt, p42/44 and β-actin was used for equal loading. Blots are representative of two independently run experiments.

To examine the effect of GSNO on growth factor induced signaling, serum starved A2780 and SKOV3 cells were treated with GSNO followed by HB-EGF treatment for various time periods. Cells were harvested and immunoblotted for various signaling molecules. As shown in **Figure 4**, treatment with GSNO inhibited HB-EGF induced activation of STAT3, Akt and p42/44 as evident from the decreased or lack of phosphorylation detected compared to HB-EGF treated samples. GSNO treatment also reduced the basal levels of phosphorylated form of Akt, STAT3 and p42/44 in A2780 and SKOV3 cells lines (**Fig. 4**). Inactive control (oxidized GSNO) had no effect on the growth factor induced signaling, suggesting the specificity of GSNO in attenuating HB-EGF induced signaling and a possible mechanism by which GSNO mediates attenuation of cell growth, migration and invasion of OvCa cells *in vitro*.

### Oral administration of GSNO attenuates tumor growth and enhances the cisplatin induced cytotoxicity *in vivo*

**Figure 5:**
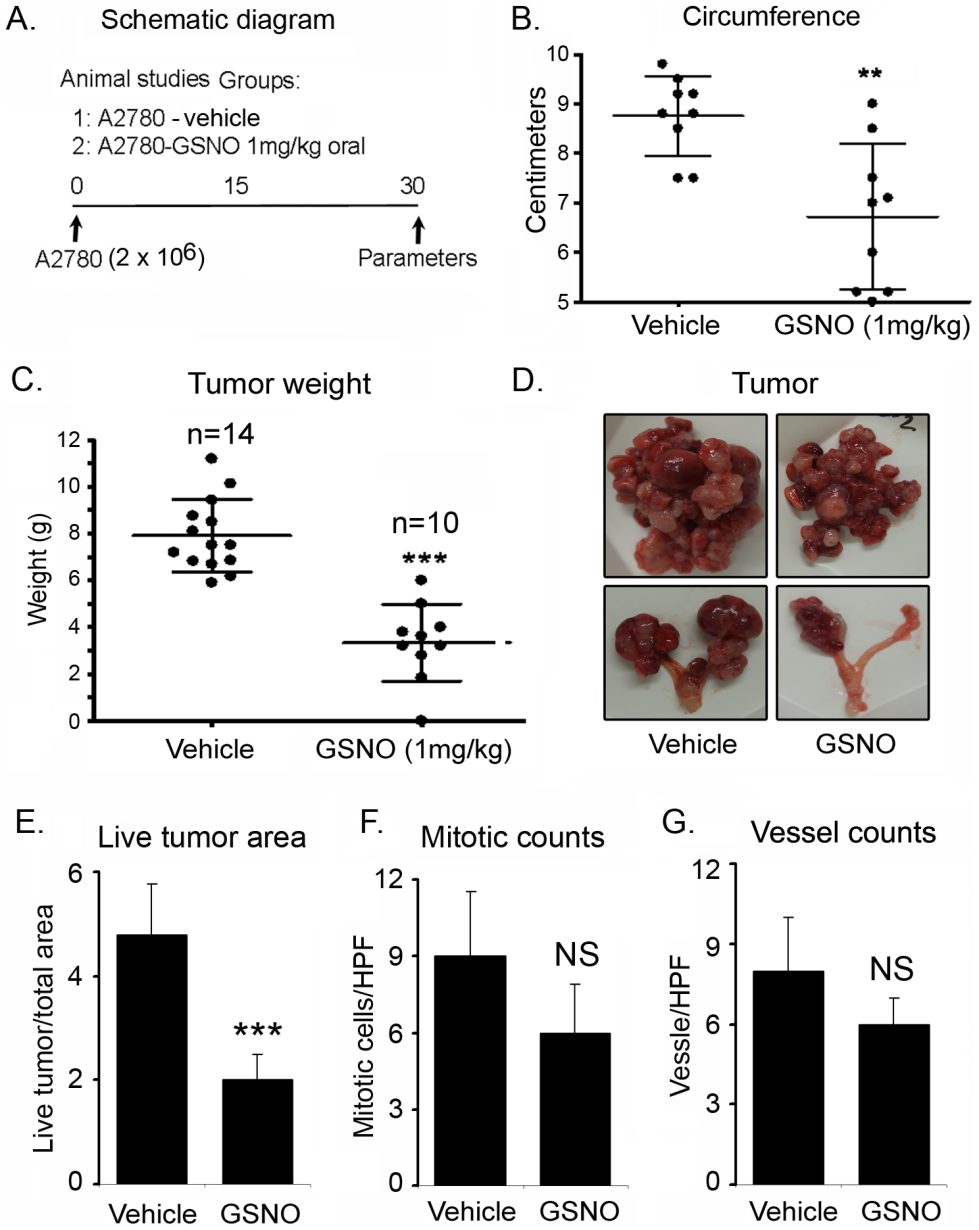
Oral administration of GSNO abrogates tumor growth in A2780 bearing nude mice. **A**. Schematic diagram of experimental design. In brief, A2780 ovarian cancer cell line was injected interperitoneally in nude mice and S-nitrosoglutathione (GSNO) was given orally daily from day 3 at the dose of 1 mg/kg of body weight till the end of the study. Phosphate buffered saline (PBS) was given as vehicle. **B**. Decreased abdominal circumference of GSNO treated mice compared to vehicle treated groups measured at week 4 (**p<0.01 treated compared to vehicle). **C.** Decreased excised tumor weights of GSNO treated mice compared to vehicle treated at week 4 (***p<0.001 treated compared to vehicle). Results are shown as mean ± SD of 10–14 individual animals. **D**. Representative gross morphological picture of tumor mass and tumor associated with ovary of vehicle and GSNO treated mice. **E**. Measurements of viable tumor size of GSNO vs PBS treated mice, as described in methods (***p<0.001 treated compared to vehicle). **F.** Count of mitotic cells and **G.** count of CD31 positive vessels per HPF (x400) in GSNO vs PBS treated mice, as described in methods), counts were performed from 5 fields of 3 different tumors from each group. NS; non-significant treated compared to vehicle.

In order to determine if GSNO could attenuate tumor growth *in vivo*, A2780 cells (2 ×10^6^) in PBS were inoculated intraperitoneally in 6-week old female nude mice for establishment of tumors. Mice were assigned to two treatment groups. The first group (untreated) was given 100 µl of PBS as vehicle by gavage. The second group was given GSNO orally in PBS (100 µl) at the dose of 1 mg/kg of body weight every day as described in the material and methods (**Fig. 5A**). At the end of 4 weeks, mice were sacrificed, and the tumor burden was grossly examined, excised and weighed. Daily administration of GSNO significantly reduced the abdominal circumference (p<0.01) (**Fig. 5B**), an indication of the tumor burden being carried in the peritoneum. GSNO also reduced tumor growth (p<0.001) in the peritoneal cavity of A2780 bearing nude mice compared to the vehicle (PBS) treated group. The mean weights of the excised tumors were approximately 68% less in GSNO (3.35±0.52 gm) treated mice compared to untreated mice (7.91±0.412 gm) (**Fig. 5C**). As previously reported by Shaw et al [12] and us [4], intraperitoneally injected A2780 cells formed mainly solid tumors and presented with ovary-specific metastases (**Fig 5D**, bottom panel). The ovary-associated tumor masses in the GSNO treated mice were much smaller than in the untreated mice (**Fig. 5D**). To further assess the viability of the tumor, necrotic and viable tumor size was determined from hematoxylin and eosin stained sections. The ratio of live tumor size to total tumor size was calculated based on the largest unidimensional diameter as described in the methods. As shown in **Figure 5E** **,** xenografts derived from GSNO treated mice had significantly less viable tumor sizes (p<0.001) and increased necrotic regions compared to xenografts derived from untreated mice. Although there was a decreasing trend observed in mitotic and vessel counts of the GSNO treated mice tumors, we could not detect a significant change (**Fig. 5F–G**). Overall, our study strongly suggests the potential of nitrosylating agent in mitigating the growth of OvCa *in vivo* (**Fig. 5**).

**Figure 6:**
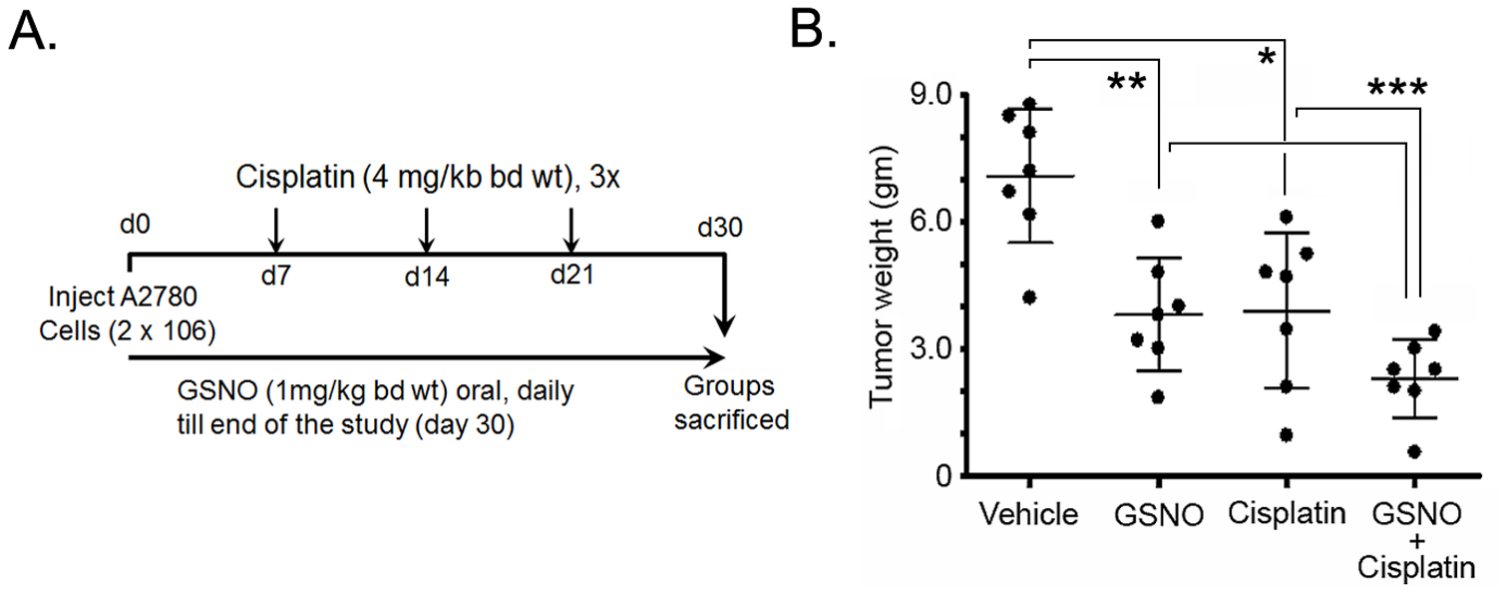
GSNO potentiates cisplatin induced cytotoxicity in A2780 bearing nude mice. **A**. Schematic diagram of experimental design. **B**. Cumulative excised tumor weight from individual mice at 4 weeks with S-nitrosoglutathione (GSNO; 1 mg/kg of body weight) (panel 2), cisplatin (4 mg/kg of body weight) (panel 3) and GSNO (1 mg/kg of body weight) and cisplatin (4 mg/kg of body weight) combination (panel 4). Cisplatin was given 3 times by intraperitoneal route at day 7, 14 and 21 post-tumor injections. Results are shown as mean ± SD of 7 individual animals. ***p<0.001 combination of GSNO + cisplatin treated group compared to untreated or cisplatin alone treated group; **p<0.01 GSNO treated group compared to untreated group; *p<0.05 cisplatin treated group compared to untreated group; #p<0.05 combination of GSNO + cisplatin group compared to GSNO alone group.

We further examined if a combination of GSNO with cisplatin could enhance the cytotoxic effect in tumors. Therefore, following the injection of A2780 cells, 2 sets of mice were treated daily with oral administration of GSNO (1 mg/kg of body weight) and cisplatin (weekly intraperitoneal injection of cisplatin on days 7, 14 and 21) as monotherapy. For combination therapy, one set of mice was treated with GSNO and cisplatin as depicted in **Figure 6A**. Both GSNO and cisplatin were significantly effective in reducing tumor growth and mass as monotherapy in A2780 bearing mice (**Fig. 6B**). However, combination therapy was more effective in inhibiting the tumor growth compared to monotherapy of either drug. Combination of cisplatin (4 mg/kg of body weight) with GSNO (1 mg/kg of body weight) (2.08±0.18 gm) (**Fig. 6B**) significantly reduced tumor growth compared to either GSNO (3.54±0.35 gm) or cisplatin treatment alone (3.15±0.34 gm) or the untreated mice (6.9±0.66 gm). Tumor volume was reduced by ∼90% in most of the mice treated with the combination. Collectively, these results suggest that combining GSNO with cisplatin treatment is significantly effective in reducing tumor growth in an OvCa mouse model.

### GSNO treatment induced nitrosylation of STAT3

Since, GSNO is a nitrosylating agent and known to mediate the posttranslational process of S-nitrosylation on proteins, which results in modulation of their activity [13]. We examined the effect of GSNO on nitrosylation on various endogenous proteins that are known to be nitrosylated and key players in OvCa including Akt, p65 and EGFR [14]–[17].

**Figure 7:**
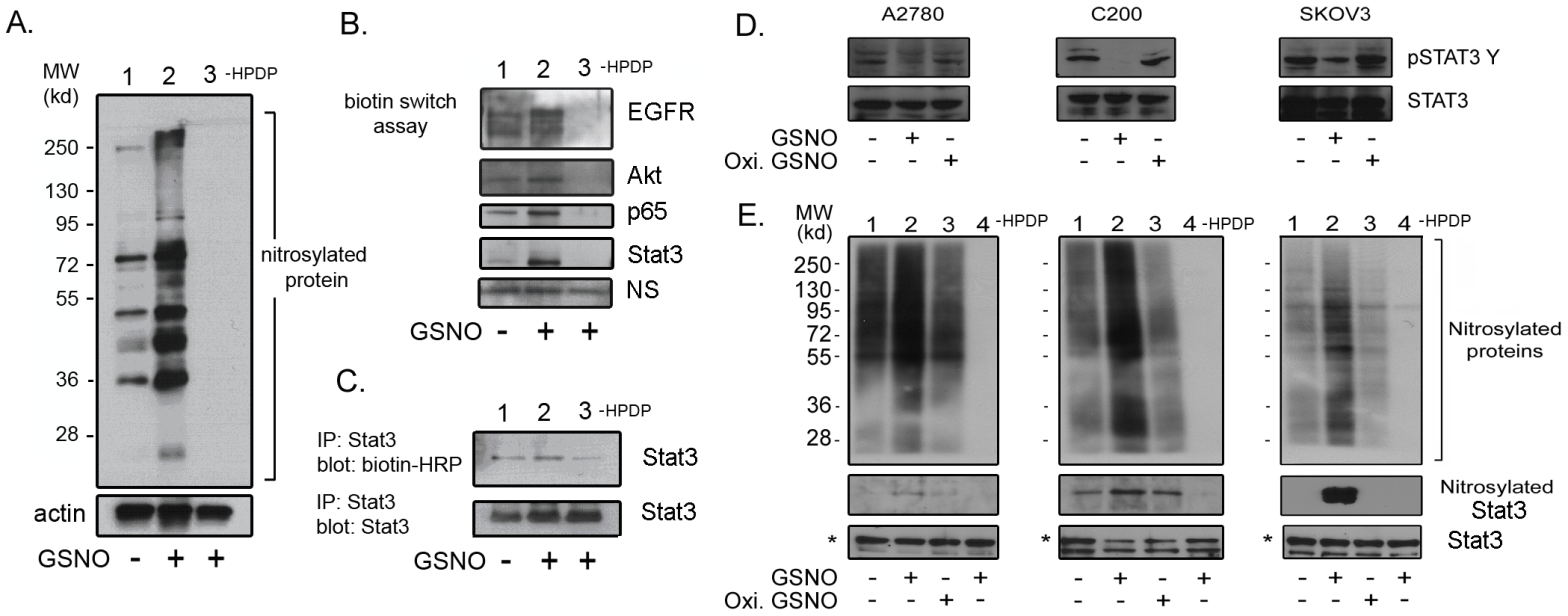
Detection of STAT3 as S-nitrosylating protein by biotin switch method. **A**. Detection of biotinylated proteins after biotin-switch method in the presence or absence of S-nitrosoglutathione (GSNO; 0.5 mM). Omission of biotin-HPRT indicates the specificity of S-nitrosylation by biotin-switch method. **B**. Detection of nitrosylation of various proteins including EGFR, p65, Akt and STAT3 in ovarian cancer cells upon GSNO treatment. **C**. STAT3 was immunoprecipitated from nitrosylated protein lysate after biotin switch assay and its biotinylation was detected by anti-biotin-HRP using western blot analysis. **D**. A2780, C200 and SKOV3 cell lines were treated with GSNO (0.5 mM) or oxidized GSNO (0.5 mM) as control for 2 hours followed by immunoblot analysis for detection of tyrosine phosphorylation of STAT3 at 705 residue and total STAT3. **E**. Under similar experimental conditions as “D”, A2780, C200 and SKOV3 cells were processed for biotin switch method for detection of STAT3. Sample in lane 4 was treated with GSNO as lane 2, except during processing for biotin switch assay; the addition of HPDP was omitted. Upper panel shows the biotinylated proteins after biotin-switch assay. Nitrosylated STAT3 was detected by pulling down biotinylated proteins by streptavidin agarose followed by immunoblot analysis using anti-STAT3 antibody. Lower bands show the total levels of input STAT3 processed for biotin switch assay.

To examine the nitrosylation of various proteins, we employed a sophisticated biotin-switch assay. Basal level of nitrosylated proteins could be detected ranging from 250 to 35 kd molecular weight in OvCa cells (**Fig. 7A** **, lane 1**). GSNO treatment potentiated the nitrosylation of endogenous proteins in treated cells with GSNO (0.5 mM) for 2 hours (**Fig. 7A** **, lane 2**). The specificity of nitrosylation could be achieved by omitting the addition of HPDP-biotin during biotin-switch assay. As shown in **Figure 7A** **, lane 3**, the omission of the HPDP-biotin step completely blocked the detection of nitrosylation of endogenous proteins, underlining the specificity of nitrosylation method in our laboratory. We further validated the induction of nitrosylation by GSNO by observing for nitrosylation of already reported signaling proteins associated with cancer progression, including ovarian. GSNO treatment induced the nitrosylation of p65, Akt and EGFR as expected (**Fig. 7B**). We further detected the unique observation of STAT3 undergoing nitrosylation upon GSNO treatment (**Fig. 7B, C**). To confirm that STAT3 is nitrosylated, STAT3 was immunoprecipitated after biotin-switch assay using its specific antibody and immunoblotted with anti-biotin-HRP. A similar observation of STAT3 undergoing nitrosylation upon GSNO treatment was seen. These sets of experiments clearly indicate a novel regulation of STAT3 by s-nitrosylation. To further examine if this phenomenon is not just confined to one cell type, we treated various OvCa cell lines including A2780, C200 and SKOV3 with GSNO and oxidized GSNO as a control. After 2 hours of incubation, total cell lysate was processed for the detection of phosphorylation status of STAT3. As shown in **Figure 7D**, GSNO treatment abolished or reduced the tyrosine phosphorylation in all OvCa cell lines without affecting the STAT3 levels. Oxidized GSNO did not affect the pSTAT3 levels in any cell lines. Under similar experimental conditions, we examined the nitrosylation of STAT3 using biotin switch method. GSNO treatment increased the total nitrosylation of multiple proteins with low to high molecular weights compared to untreated and oxidized GSNO treated samples as evident from **Figure 7E**. A similar pattern was observed in nitrosylation of STAT3 suggesting that the GSNO mediated increase in nitrosylation of STAT3 is a general phenomenon in all OvCa cell lines.

### GSNO nitrosylates STAT3 and affects its ability to bind DNA

**Figure 8:**
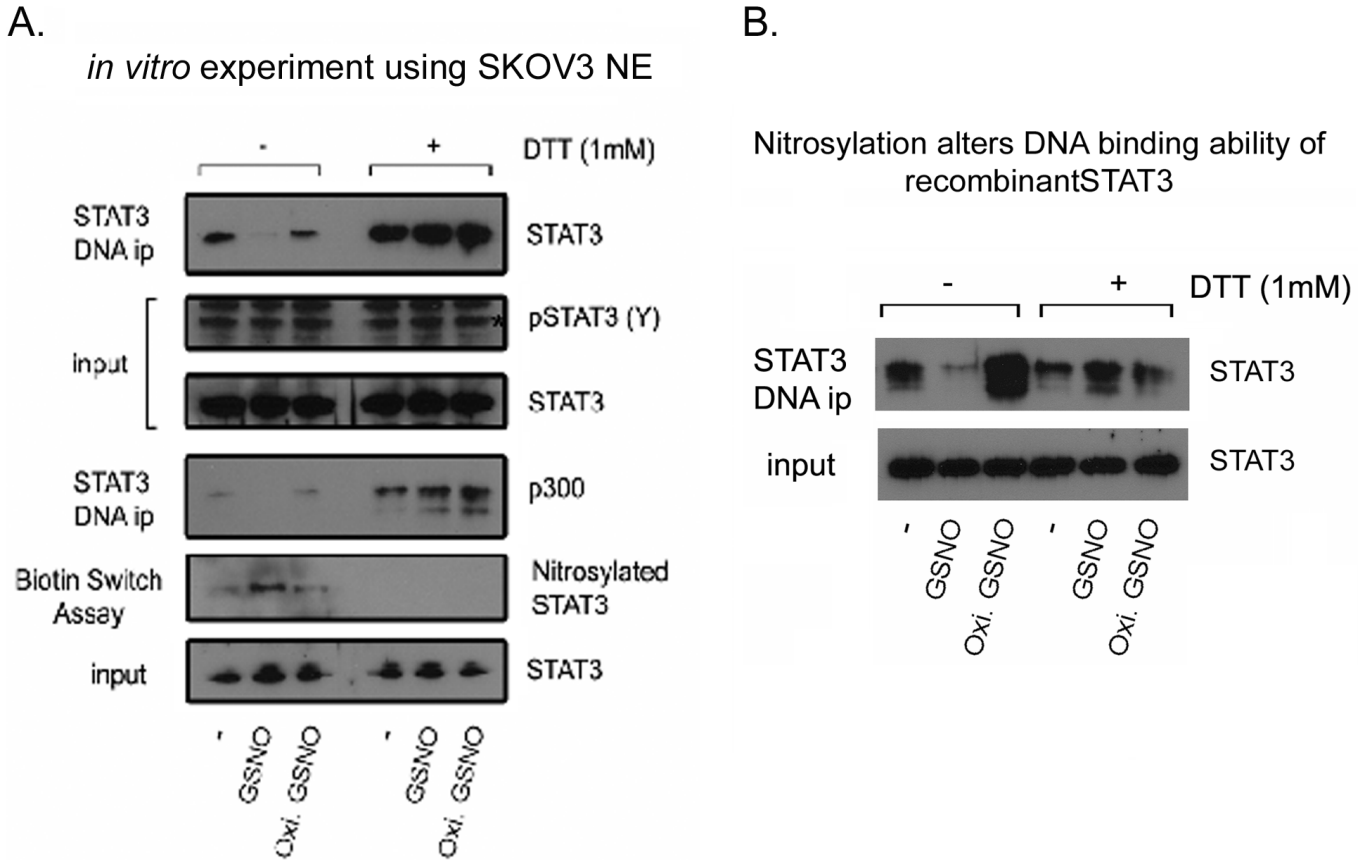
*In vitro* nitrosylation of STAT3 affects its DNA binding ability. **A**. Nuclear extract (NE) from SKOV3 was isolated and 20 µg was incubated with S-nitrosoglutathione (GSNO) or oxidized GSNO (100 µM) at 4°C in the presence or absence of DTT (1 mM). After 4 hours of incubation, DNA binding ability of nuclear STAT3 was examined by incubating nitrosylated NE with STAT3 gel shift oligonucleotides conjugated with agarose followed by immunoblot analysis with anti-STAT3 antibody. Since STAT3 recruits coactivator, including p300, we also examined the recruitment of p300 under similar experimental conditions. STAT3 and pSTAT3 (Y705) were examined before pulling down STAT3 with gel shift oligonucleotides conjugated with agarose. Nitrosylation of STAT3 was also examined under similar experimental conditions using biotin switch method. Total STAT3 level was examined in the input of NE from SKOV3, which was used to show that an equal protein amount was used in this experiment. **B**. Recombinant STAT3 (1 µg) was incubated with GSNO or oxidized GSNO (0.1 mM) at 4°C in the presence or absence of DTT (1 mM). After 2 hours of incubation, DNA binding ability of recombinant STAT3 is examined by pulling down Stat3 by incubating with gel shift oligonucleotides conjugated with agarose.

Once we established that STAT3 undergoes nitrosylation in cultured cells, we further examined if STAT3 could be nitrosylated *in vitro* and if it may affect its DNA binding ability. For this, we took two approaches, first we isolated nuclear extract from SKOV3, which exhibits higher basal levels of the phosphorylated form of STAT3 and has the ability to bind STAT3 DNA binding motif without stimulation. We incubated 20 µg of nuclear extract (NE) with GSNO or oxidized GSNO in the presence or absence of DTT for 4 hours. Following incubation, the DNA binding ability of STAT3 was examined by incubating nitrosylated NE with STAT3 gel shift oligonucleotides conjugated with agarose. As shown in **Figure 8A**, the GSNO treated sample showed nitrosylation of STAT3 as evident by biotin switch assay and could not be pulled down by STAT3 gel shift oligonucleotides conjugated with agarose. Inclusion of DTT in samples abolished GSNO mediated nitrosylation of STAT3, resulting in the binding of STAT3 on its DNA binding motif. STAT3 also has been shown to recruit coactivator including p300. We also found that while untreated NE showed recruitment of p300, nitrosylated STAT3 (GSNO treatment) could recruit p300 (**Fig. 8A**), further suggesting the role of nitrosylation in regulating gene transcription in general. As a second approach, we used recombinant STAT3 protein. We nitrosylated recombinant STAT3 for 2 hours with GSNO and pulled down with STAT3 gel shift oligonucleotides conjugated with agarose (**Fig. 8B**). Untreated STAT3 was able to bind to the DNA binding motif and could be observed after pull down; however, GSNO treated STAT3 did not bind, and was not seen bound to the DNA. Treatment with DTT reversed the GSNO mediated inability of recombinant STAT3 to bind DNA motif (**Fig. 8B**), further strengthening the point that STAT3 could be nitrosylated *in vivo* and *in vitro*, which could affect its ability to bind STAT3 DNA motif.

## Discussion

The combination of cytoreproductive surgery with cisplatin-centered chemotherapy has significantly enhanced the survival of patients and reduced the mortality of OvCa, but development of chemoresistance and relapsing of OvCa in treated patients is still responsible for the low survival rates. The current major focus is to identify an effective therapeutic treatment which not only reduces tumor mass, but also potentiates the chemotherapy outcome. In the present study we examined the therapeutic potential of a nitrosylating agent, GSNO, using cell culture based and an *in vivo* preclinical mouse model of OvCa studies. We show that GSNO treatment attenuated cell growth of various chemoresistant and chemosensitive OvCa cell lines including A2780, C200, SKVO3, ID8, OVCAR3, OVCAR4, OVCAR5, OVCAR7, OVCAR8, OVCAR10, PE01 and PE04. It also inhibited growth factors induced activation of STAT3, Akt and p42/44 resulting in attenuation of cell invasion and migration of OvCa. Daily oral administration of GSNO at the dose of 1 mg/kg of body weight was not only able to reduce tumor burden, but also enhanced the cisplatin mediated cytotoxic effect in nude mice bearing human ovarian xenografts. Being a nitrosylating agent, GSNO treatment induced the nitrosylation of various known proteins including NFκB p65, Akt and EGFR. As a novel finding, we found, GSNO also induced nitrosylation of STAT3, which is a known player in chemoresistance and cell proliferation in OvCa and in cancer in general. Overall, our study presents a novel attribute of a nitrosylating agent, like GSNO, as a tumor suppressor agent, which can be used as a therapeutic in ovarian and other cancers.

NO has been shown to be cytotoxic for tumor cells [18]. NO could be produced in tumors by inducible nitric oxide synthase (iNOS), a pro-inflammatory mediator which is expressed under inflammatory and pathological conditions. Expression of iNOS has been reported in human ovarian tumors [18]–[20]. Exogenous NO donors, including S-nitroso-N-acetyl-penicillamine (SNAP) and sodium nitroprusside (SNP), have been shown to induce cell death by down regulating the surviving network in OvCa [21]. These NO donors are fast acting and spontaneously release NO in aqueous media irrespective of the presence or absence of cells. In contrast, GSNO being a natural compound releases NO in a slow defined manner. Uncontrolled delivery of NO could not only affect tumor cells, but could also be deleterious to stromal normal cells. Therefore, use of controlled NO releasing molecules seems to be a better approach. Under slow release NO donors, including NO releasing silica nanoparticle and NO-releasing aspirin, have been used in OvCa [22]–[24]. Our finding shows that slow releasing NO from GSNO attenuated the growth of various OvCa cell lines; however, oxidized GSNO (not able to produce NO) did not show any effect on cell proliferation. Similar to our data, a previous study using NO releasing silica nanoparticles also inhibited anchorage-independent growth of tumor-derived and Ras-transformed OvCa using a cell culture based study [22]. NO releasing aspirin (NCX-4040) has also been shown to attenuate cisplatin-sensitive and cisplatin-resistant OvCa cells *in vivo* and *in vitro* [23], [24]. Our results are inconsistent with these findings as GSNO was able to inhibit growth of chemoresistant and chemosensitive OvCa cell lines. GSNO also inhibited activation of EGFR and STAT3 in OvCa, which is consistent with the previous finding using NCX-4040; however, this report did not show the mechanism. Moreover, a higher dose of NCX-4040 (50-100 mg/kg of body weight) was required to show effectiveness in tumor regression in ovarian tumor bearing mice and was given intraperitoneally. However, oral administration of GSNO at the dose of 1 mg/kg of body weight was significantly effective in reducing tumor burden in nude mice bearing human A2780 carcinoma. GSNO is a naturally-occurring compound in the body that slowly releases NO under physiological conditions, and its main mode of action has been shown to nitrosylate a number of proteins involved in diverse signaling including cell proliferation, migration and chemo resistance.

Protein nitrosylation is a reversible posttranslational modification in which NO moieties are covalently bound to reactive cysteine thiols via the S-nitrosothiol bond. S-nitrosylation has been reported to have a profound effect on several biological molecules involved in normal and pathological signaling in cells, including Akt [14], NFκB [15], [16], IKKbeta [25], PTEN [26], phosphatase, EGFR [17] and caspase 3 [27]. GSNO has been shown to provide a cytoprotective effect in ischemia reperfusion injury [28], hemorrhagic shock [29] and in models of autoimmune diseases [16], [30]. We report for the first time GSNO mediated nitrosylation of various proteins in OvCa including EGFR, Akt, NFκB p65 and STAT3. NO mediated inactivation of EGFR has been reported previously [31]; however, its nitrosylation and nitrosylation sites (C166 and C305) have only been identified recently [17], [32]. Our findings of the loss of phosphorylation of EGFR in response to GSNO treatment and the enhancement of nitrosylation in OvCa are in accordance with these reports. We also observed an increase in the nitrosylation of Akt and p65 in GSNO treated OvCa cells. Akt1 is also nitrosylated at the residue C224 and is inactivated under pathogenesis of iNOS and/or oxidative stress-involved insulin resistance [14]. The p65, an important component of the NFκB pathway, is also reported as being regulated by nitrosylation and impairs its ability to bind NFκB consensus binding site(s) in promoters of NFκB regulated genes [15], [16], [33], [34].

GSNO treatment not only attenuated growth factors mediated activation of signaling molecules involved in cell proliferation including Akt, p42/44 and STAT3 but also inhibited their basal level in A2780 and SKOV3. Abnormal activation of Akt is well documented in human ovarian cancer and play important role in ovarian carcinogenesis [35], [36]. Consistent with these reports, the basal activity of Akt was found to be high in A2780 which was completely attenuated by GSNO treatment. In SKOV3 cell line, STAT3 is highly activated at basal level due to over expression of activated EGFR [37]. GSNO treatment also attenuated phosphorylation of STAT3 in SKOV3 in the presence or absence of growth factor. GSNO mediated inhibition of activated Akt and STAT3 in A2780 and SKOV3, respectively, might be responsible for attenuation of cell proliferation in these cell lines as well as other OvCa cell lines used in this study. Attenuation of growth factor induced signaling by GSNO treatment in OvCa cell lines is directly reflecting by the inhibition of cell proliferation in these cell lines. Our data clearly showed that nitrosylation could be one of the important modifications leading the abrogation of phosphorylation of various signaling molecules including EGFR, Akt and STAT3.

Signal transducers and activators of transcription (STATs) are a group of transcription factors that have been increasingly implicated in cancer pathogenesis [38], [39]. Of the 7 known STAT proteins, activated STAT3 is reported to be strongly associated with various cancers including OvCa [40]–[45]. Constitutive activation of STAT3 signaling is observed in a number of neoplasms, including acute leukemia [46], breast cancer [47], squamous cell carcinoma of the head and neck [48], multiple myeloma [49] and OvCa [40]–[45]. In OvCa, increased STAT3 directed transcription has been implicated in the stimulation of proliferation seen in response to cytokines, including VEGF and IL-6, in invasiveness [44] and as a predictor of poor prognosis [50]. It activates the transcription of a number of genes, including antiapoptotic proteins Bcl-2, Bcl-x_L_ and Mcl-1 [39], [51], [52]. Moreover, constitutive activation of the STAT3 pathway has recently been shown to confer resistance to chemotherapy-induced apoptosis in epithelial malignancies [53]–[56]. Because of the vast data implicating STAT3 activation in cancer pathogenesis, inhibition of the STAT3 signaling pathway has tremendous implications in the treatment as well as reversing the drug resistance seen in OvCa patients [38], [39], [57]–[65]. The activity of STAT3 and its interaction with other proteins are modulated by phosphorylation [66], [67] and acetylation [68], but other posttranslational modifications, including S nitrosylation, are not reported. Our findings report nitrosylation as a novel modification of STAT3 in GSNO treated OvCa cells. Using biotin switch method, basal level of nitrosylated STAT3 was detected and GSNO treatment enhanced it many folds. This observation was confirmed by detection of biotinylated STAT3 using immunoblot after biotin switch assay followed by immunoprecipitation of STAT3. Currently nitrosylation site(s) in STAT3 is not known. Identification of nitrosylation site(s) in STAT3 and their regulation will lead to a better understanding of the exact underlying biology of STAT3 in the pathogenesis of OvCa.

Overall, our study highlights an important role of nitrosylation as a regulatory process involved in modulation of various proteins in OvCa. Our data also clearly indicates the therapeutic potential of nitrosylating agents like GSNO as a mono-therapy or in combination with chemotherapy for the treatment of OvCa and could modulate a number of oncogenes like EGFR, STAT3, NFκB (p65), Akt and IKK. Therefore, this approach follows the new strategy of “one-drug, multiple-target” therapy in OvCa.

## Supporting Information

Figure S1 **GSNO mediated release of NO in the medium containing A2780 or SKOV3 cells.** **A**. GSNO solution (0.2 mM in DMSO; pink in color) was exposed to light for 7 days to prepare inactive oxidized GSNO (colorless). **B**. A2780 and SKOV3 cells were plated in 24 well plates at the density of 50×10^3^ cells/well. After 24 h incubation, cells were treated with various concentrations of GSNO ranging from 0.01 to 1 mM. After 24 h of treatment, NO was measured in medium with Griess reagent. **C**. NO was measured in medium in oxidized GSNO and GSNO treated A2780 and SKOV3 cells at 24 hour (N = 4).
(TIF)

Figure S2 **GSNO treatment attenuated cell proliferation of multiple ovarian cancer cell lines. A-F.** Various ovarian cancer cell lines including OVCAR-3, -4, 5, -7, -8 and -10 were treated with various concentration of GSNO (0.1-1 mM). Percent viability of these cell lines were determined by MTT assay after 48 h of treatment. Inactive GSNO (oxidized, last bar) was used as control. The data is represents three individual experiments done in triplicates. ***p< 0.001; **p< 0.01; *p< 0.05 and NS; not significant compared to untreated cells using Student’s t-test (Prism).
(TIF)

Table S1 **Measurement of IC50 of GSNO mediated effect on cell proliferation of human ovarian cancer (OvCa) cell lines.** OvCa cell lines were treated with various concentrations of GSNO and cell viability was measured by MTT. IC 50 was calculated using CalcuSyn software (Biosoft, Cambridge, UK). Values are presented as mean ± SD of three values.
(DOCX)

Table S2 **Measurement of IC 50 of GSNO mediated effect on clonogenic survival of human ovarian cancer (OvCa) cell lines.** OvCa cells (2×10^3^) were plated in triplicates in 6-well plate and after 24 hour, cells were treated with indicated concentrations of GSNO once. The cells were allowed to form colonies for up to 2 weeks. Colonies were stained with MTT, counted and IC 50 was calculated using CalcuSyn software (Biosoft, Cambridge, UK). Values are presented as mean ± SD of three values.
(DOCX)
