# Supplementary figures and images for: Preclinical Therapeutic Potential of a Nitrosylating Agent in the Treatment of Ovarian Cancer
Source: PLoS One. 2014 Jun 2;9(6):e97897. doi: 10.1371/journal.pone.0097897 (PMC4041717; doi:10.1371/journal.pone.0097897)

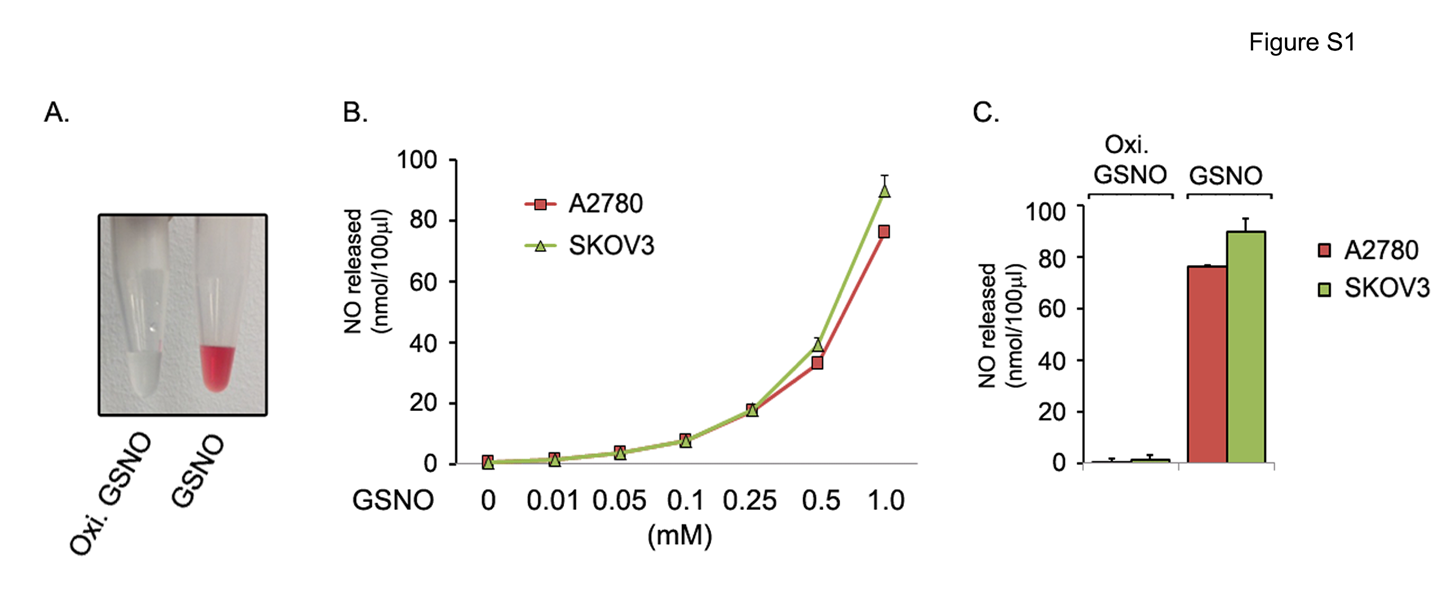

Supplement: Figure S1 — GSNO mediated release of NO in the medium containing A2780 or SKOV3 cells. A. GSNO solution (0.2 mM in DMSO; pink in color) was exposed to light for 7 days to prepare inactive oxidized GSNO (colorless). B. A2780 and SKOV3 cells were plated in 24 well plates at the density of 50×103 cells/well. After 24 h incubation, cells were treated with various concentrations of GSNO ranging from 0.01 to 1 mM. After 24 h of treatment, NO was measured in medium with Griess reagent. C. NO was measured in medium in oxidized GSNO and GSNO treated A2780 and SKOV3 cells at 24 hour (N = 4). (TIF) [file pone.0097897.s001.tif]

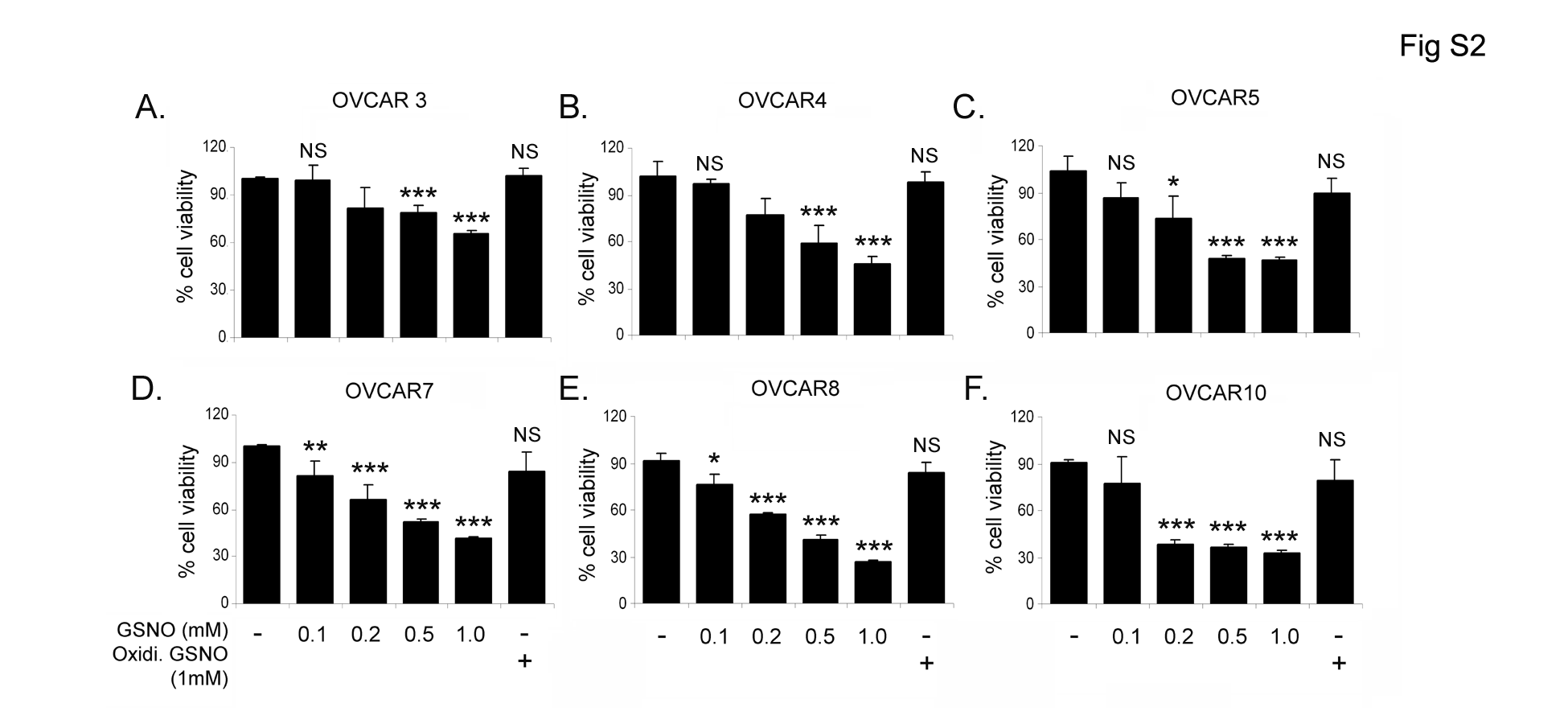

Supplement: Figure S2 — GSNO treatment attenuated cell proliferation of multiple ovarian cancer cell lines. A-F. Various ovarian cancer cell lines including OVCAR-3, -4, 5, -7, -8 and -10 were treated with various concentration of GSNO (0.1-1 mM). Percent viability of these cell lines were determined by MTT assay after 48 h of treatment. Inactive GSNO (oxidized, last bar) was used as control. The data is represents three individual experiments done in triplicates. ***p< 0.001; **p< 0.01; *p< 0.05 and NS; not significant compared to untreated cells using Student’s t-test (Prism). (TIF) [file pone.0097897.s002.tif]
