# Supplementary material for: Preclinical Therapeutic Potential of a Nitrosylating Agent in the Treatment of Ovarian Cancer
Source: PLoS One. 2014 Jun 2;9(6):e97897. doi: 10.1371/journal.pone.0097897 (PMC4041717; doi:10.1371/journal.pone.0097897)
Supplement: Table S1 — Measurement of IC50 of GSNO mediated effect on cell proliferation of human ovarian cancer (OvCa) cell lines. OvCa cell lines were treated with various concentrations of GSNO and cell viability was measured by MTT. IC 50 was calculated using CalcuSyn software (Biosoft, Cambridge, UK). Values are presented as mean ± SD of three values. (DOCX) [file pone.0097897.s003.docx]

| **OvCa cell lines** | **IC50 (mmol/L)** |
| --- | --- |
| A2780 | 162.64 + 25 |
| C200 | 385.32 + 50 |
| PE01 | 337.75 + 39 |
| PE04 | 426.16 + 72 |
| OV2O2 | 196.06 + 34 |
| SKOV3 | 235.53 + 20 |
| OVCAR3 | 3463.21 + 293 |
| OVCAR 4 | 1680.96 + 201 |
| OVCAR 5 | 766.73 + 36 |
| OVCAR 7 | 709.24 + 59 |
| OVCAR 8 | 536.34 + 37 |
| OVCAR 10 | 363.43 + 41 |

**Table S1:** Measurement of IC50 of GSNO mediated effect on cell proliferation of human ovarian cancer (OvCa) cell lines.

Values are presented as mean + SD of three values.
