# Supplementary material for: Preclinical Therapeutic Potential of a Nitrosylating Agent in the Treatment of Ovarian Cancer
Source: PLoS One. 2014 Jun 2;9(6):e97897. doi: 10.1371/journal.pone.0097897 (PMC4041717; doi:10.1371/journal.pone.0097897)
Supplement: Table S2 — Measurement of IC 50 of GSNO mediated effect on clonogenic survival of human ovarian cancer (OvCa) cell lines. OvCa cells (2×10^3^) were plated in triplicates in 6-well plate and after 24 hour, cells were treated with indicated concentrations of GSNO once. The cells were allowed to form colonies for up to 2 weeks. Colonies were stained with MTT, counted and IC 50 was calculated using CalcuSyn software (Biosoft, Cambridge, UK). Values are presented as mean ± SD of three values. (DOCX) [file pone.0097897.s004.docx]

**Table S2**: Measurement of IC50 of GSNO mediated effect on clonogenic survival of human ovarian cancer (OvCa) cell lines.

Values are presented as mean + SD of three values.

| **OvCa cell lines** | **IC50 (mmol/L)** |
| --- | --- |
| A2780 | 122.9 + 27 |
| C200 | 161.57 + 20 |
| PE01 | 356.24 + 30 |
| PE04 | 353.25 + 50 |
